# A randomized double-blinded study assessing the effect of different doses of transnasal dexmedetomidine on the median effective concentration of ropivacaine for a caudal block

**DOI:** 10.3389/fmed.2024.1481938

**Published:** 2024-11-18

**Authors:** Fu Wang, Shijie Qu, Yinglu Chen, Bo Liao, Li Ao, Hui Zhang, Hongyan Zhou, Liang Zhang

**Affiliations:** ^1^Department of Anesthesiology, Chongqing Traditional Chinese Medicine Hospital, Chongqing, China; ^2^Department of Proctology, Chongqing Traditional Chinese Medicine Hospital, Chongqing, China

**Keywords:** caudal block, EC50, ropivacaine, intranasal dexmedetomidine, hemorrhoidectomy

## Abstract

**Background:**

Perineural administration of dexmedetomidine (PN-DEX) can enhance the efficacy of local anesthetics used in regional nerve blocks while decreasing the median effective concentration (EC50) of these anesthetics. Intranasal administration of dexmedetomidine (IN-DEX) is more accessible for sedation during regional anesthesia because of its non-invasive systemic administration and demonstrates synergism with local anesthetic. However, it remains unclear whether IN-DEX affects the EC50 of local anesthetics used in caudal blocks.

**Methods:**

This study was a prospective, single-center, double-blind, randomized controlled trial. Patients scheduled to undergo elective hemorrhoidectomy were included and divided into three groups. Furthermore, 0.01 mL/kg of normal saline and 1 μg/kg and 2 μg/kg of dexmedetomidine were dripped into both nostrils of the patients in groups IN-NS, IN-DEX1, and IN-DEX2, respectively. These were administered 15 min before the caudal block. The initial concentration of ropivacaine was set at 0.4%, which was then varied by 0.025% using the up-and-down sequential allocation method. Vital signs, instances of hypotension and bradycardia with treatment, and other adverse reactions were recorded and compared.

**Results:**

The EC50 values of ropivacaine were 0.275% (95% confidence interval (CI), 0.254–0.296%) in group IN-NS, 0.257% (95% CI, 0.238–0.276%) in group IN-DEX1, and 0.216% (95% CI, 0.195–0.236%) in group IN-DEX2. The EC95 values of ropivacaine were 0.315% (95% CI, 0.295–0.370%) in group IN-NS, 0.297% (95% CI, 0.278–0.351%) in group IN-DEX1, and 0.256% (95% CI, 0.236–0.310%) in group IN-DEX2. Compared to group IN-NS, the EC50 value of ropivacaine in IN-DEX2 was significantly decreased by 21.4% (*p* = 0.001), while there was no significant difference between group IN-NS and IN-DEX1 (*p* = 0.125). There were no differences in hypotension and bradycardia with treatment among the different groups.

**Conclusion:**

IN-DEX decreased the EC50 of ropivacaine for the caudal block, and there was a specific dose-dependent effect for IN-DEX. The side effects were similar across all groups.

## 1 Introduction

A single-shot caudal block could be the best choice for perioperative anesthesia management in patients undergoing hemorrhoidectomy because it is easy to perform, is less traumatic, is a reliable analgesic, and provides relaxation of the anal sphincter (1). A caudal block is usually performed using a single large dose of a local anesthetic, which may lead to anesthetic intoxication and increase the risk of motor weakness, delayed micturition, or urinary retention (2, 3). Therefore, it is important to reduce the dosage of local anesthetics required by investigating the median effective concentration (EC50) of the local anesthetics.

Several adjuvants, in combination with local anesthetics, have been used to produce a synergistic effect and decrease the required concentration of local anesthetics for a caudal block. The European Society of Regional Anesthesia and Pain Therapy and the American Society of Regional Anesthesia and Pain Medicine joint committee practice advisory on pediatric regional anesthesia has recommended α2-agonists (such as clonidine and dexmedetomidine) for caudal blocks (4). Evidence suggests that dexmedetomidine may be more efficacious than clonidine (5, 6). Dexmedetomidine is a highly selective α_2_ adrenergic receptor agonist used for its sedative, analgesic, and anxiolytic properties (7). In recent years, some studies have extensively studied the effects of dexmedetomidine on the efficacy of local anesthetics and found that perineural administration of dexmedetomidine (PN-DEX) can synergistically enhance the effects of local anesthetics in peripheral nerve blocks and spinal anesthesia, including shortening the onset time of local anesthetics and prolonging the duration of analgesia (8, 9). A similar effect was also observed in caudal blocks (10, 11).

Recent studies have found that the analgesic mechanism of dexmedetomidine may not be single and that the site is not limited to the periphery (12). Some studies have shown that PN-DEX decreases the EC50 of ropivacaine or lidocaine (13, 14). Caudal dexmedetomidine has also been found to decrease the EC50 of local anesthetics for caudal blocks (15). In addition, intravenous administration of dexmedetomidine (IV-DEX) has been shown to reduce the use of local anesthetics. Our previous study also confirmed that IV-DEX can reduce the EC50 of ropivacaine for caudal blocks (16). Intranasal administration of dexmedetomidine (IN-DEX) is more accessible for sedation during regional anesthesia because of its non-invasive systemic administration (17). It is unknown whether IN-DEX could also affect the EC50 of local anesthetics for caudal blocks. The present research aimed to explore whether IN-DEX could decrease the EC50 of ropivacaine for caudal blocks.

## 2 Materials and methods

### 2.1 Ethics statement

This prospective, single-center, double-blind, randomized controlled study was approved by the ethics committee of Chongqing Traditional Chinese Medicine Hospital (approval number: 2018-ky-1) and registered at www.chictr.org.cn (ChiCTR1800015409) on 28th March 2018. Patients enrolled in this study were asked to sign a written informed consent form.

### 2.2 Patient enrollment

After obtaining the written informed consent forms and completing the study screening, patients aged 20–50 years with ASA physical status I and II undergoing elective hemorrhoidectomy with caudal blocks were included. The exclusion criteria were as follows: (1) bleeding diathesis; (2) infection at the puncture site; (3) history of central nervous system surgeries or diseases, including schizophrenia, epilepsy, Parkinsonism, and myasthenia gravis; (4) history of nasal surgeries and illnesses, including rhinitis, nasal polyp, and nasosinusitis; (5) history of local anesthetic allergy; (6) diabetes; (7) secondary anus surgery; (8) hypertension, coronary heart disease, or cardiac conduction block; and (9) body mass index >30 kg/m^2^.

### 2.3 Grouping and trial protocol

All eligible patients were randomly divided into three groups: group IN-NS (intranasally administered 0.9% of normal saline), IN-DEX1 (intranasally administered 1 μg/kg of dexmedetomidine), and IN-DEX2 (intranasally administered 2 μg/kg of dexmedetomidine). The randomization was achieved using a computationally generated random number sheet, with the numbers placed in continuously numbered opaque envelopes. The group allocation was performed by a nurse, who was not involved in data collection and patient management. We intranasally administered normal saline or dexmedetomidine (Aibeining; Jiangsu Hengrui Medicine Co., Ltd., China) 15 min before the caudal block. We prepared 0.01 mL/kg 0.9% normal saline, as well as 1 μg/kg and 2 μg/kg dexmedetomidine, using a 1-ml syringe according to the patient’s body weight. The patients lay in a supine position. We administered the drugs into both nostrils of the patients at an angle of 30° over 2 min to maximize drug absorption (18).

### 2.4 Caudal block

**Figure 1:**
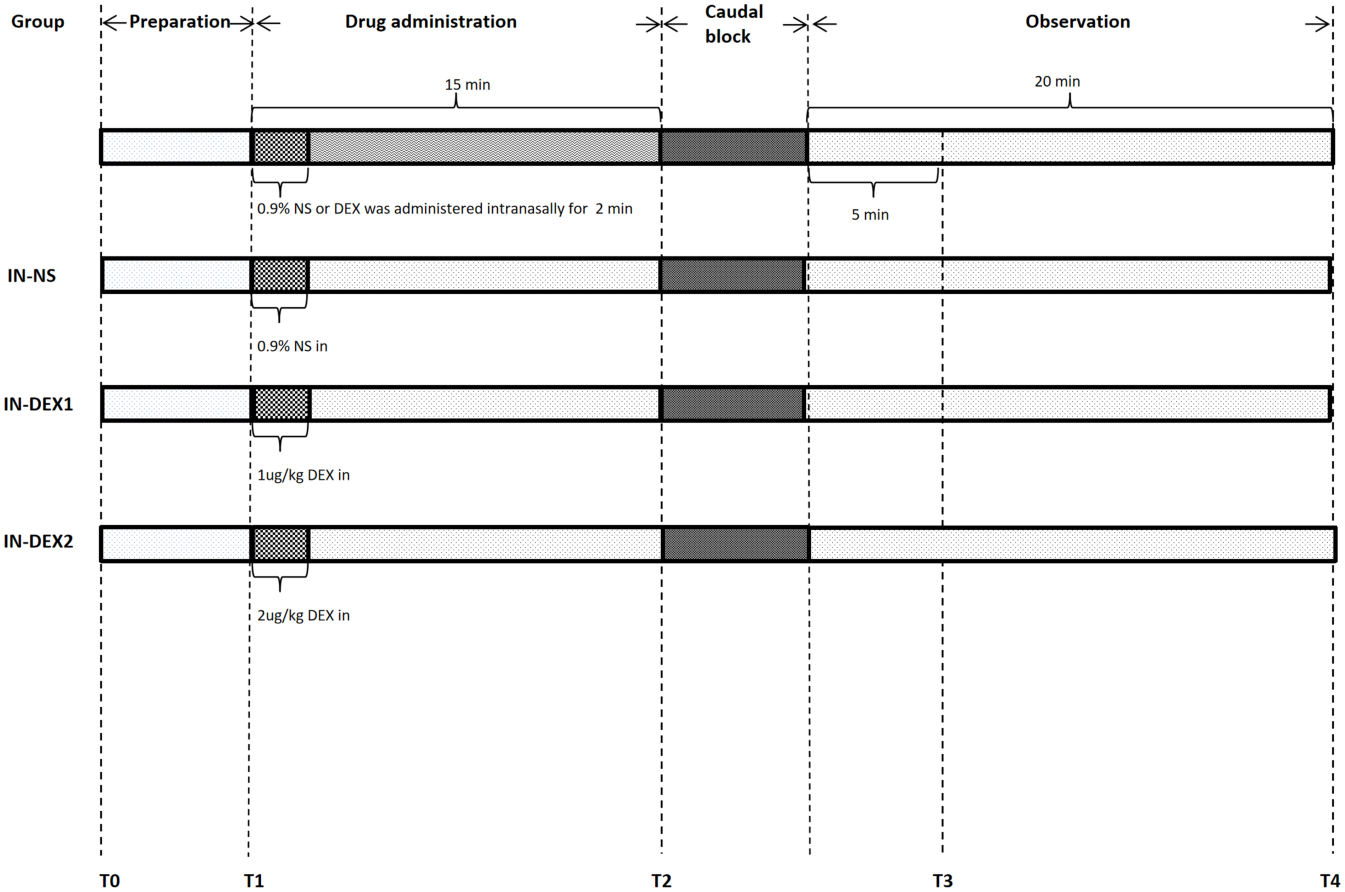
Anesthesia management flow chart. HR, SBP, and DBP were recorded and analyzed at the following time points: T0, baseline; T1, 0 min before drug adminstration; T2, 0 min before caudal block; T3, 5 min after caudal block; and T4, 20 min after caudal block.

Upon arrival in the operating room, all patients lay in a supine position and received standard monitoring, including blood pressure, heart rate, respiratory rate, electrocardiogram, and pulse oximetry. Systolic blood pressure (SBP), diastolic blood pressure (DBP), and heart rate (HR) were recorded and analyzed at the following time points (Figure 1): baseline (T0), 0 min before dexmedetomidine or normal saline administration (T1), 0 min before the caudal block (T2), 5 min after the caudal block (T3), and 20 min after the caudal block (T4). The patients were admitted to the ward with a 22G intravenous line, and 500 mL of Ringer’s lactate solution was administered intravenously. All procedures were carried out with ultrasound localization.

**Figure 2:**
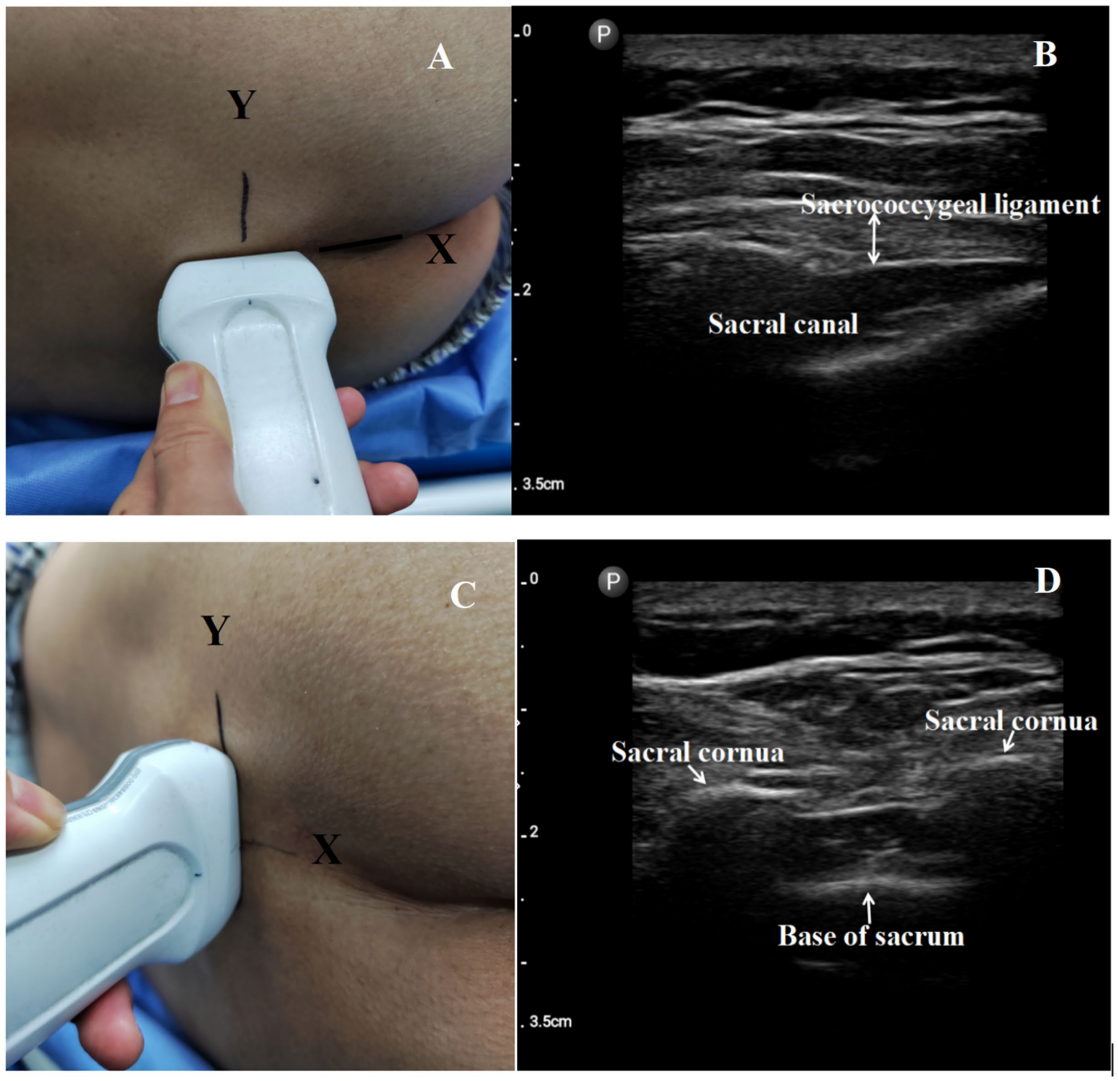
**(A)** Ultrasound positioning procedure before puncture. Place the linear array probe parallel to the long axis of the body in the approximate position, and draw a line to mark the “*x*” axis. **(B)** Longitudinal ultrasound image of the sacral canal. **(C)** Then ultrasonic probe was rotated 90 degrees to get a landscape view, and draw a line to mark the “*y*” axis. **(D)** Transversal ultrasound image of the sacral canal.

Following preparation, the patients were positioned in a left lateral position, and an experienced anesthesiologist performed the caudal epidural block. Ropivacaine (75 mg/10 mL Naropin; AstraZeneca, Sodertalje, Sweden) used for the caudal block was diluted to 20 mL with 0.9% saline according to the target concentration. All patients were positioned in a left lateral position during the caudal block. First, the approximate position (the equilateral triangle located between the apex of the sacral hiatus and the superolateral sacral crests) was determined by touch, and a “+” mark was made. Then, an ultrasound was performed to locate the exact position. The linear array probe was positioned parallel to the long axis of the body at the approximate position to identify the sacral ligament, and a line was drawn to mark the “*x*” axis (Figures 2A,B). Then, the ultrasonic probe was rotated 90 degrees to obtain a landscape view. Both sides of the sacral cornu, the sacrococcygeal ligament, and the sacral base were illustrated in the transverse view. A line was drawn to mark the “*y*” axis in this view (Figures 2C,D). The intersection point of the “*x*” axis and “*y*” axis was designated as the exact puncture point. After confirming the puncture point, a 22G needle was inserted into the caudal space under sterile conditions through the sacrococcygeal ligament. The caudal space was identified by the loss of resistance to air. After negative aspiration with no blood or cerebral spinal fluid, we injected ropivacaine slowly over 2 min. We assessed the analgesic effectiveness of the caudal block using pinprick testing of the perineal anal area at the following time points: baseline, immediately after the block, and 20 min after the caudal block. The anesthetic effects of the patients were classified as follows:

1. The caudal block was considered effective if there was no pain in response to the pinprick testing of the perineal anal area.
2. The caudal block was considered ineffective if the patient experienced numbness but still perceived pain in response to the pinprick testing of the perineal anal area.
3. The caudal block was considered a technical failure in cases of vascular puncture, local anesthetic toxicity, unilateral block, or no anesthetic effect of the caudal block in the patients.

### 2.5 Anesthesia management

For a technical failure, the patients received rescue anesthesia, which included supplemental opioids, local infiltration anesthesia by the surgeon, or another caudal block. We administered atropine intravenously to treat bradycardia (<50 bpm). Hypotension (SBP < 90 mmHg or a decrease of 20% from baseline) was managed with intravenous ephedrine and fluid infusion. Any other adverse effects after the anesthesia were recorded, such as shivering, pruritus, respiratory depression, nausea, and vomiting.

### 2.6 Blinding method

All blocks were performed by one experienced anesthetist using the same ultrasound probe (Philips, Lumify L12-4, United States). A nurse, who did not participate in follow-up research, prepared the local anesthetics based on the responses of the previous patients. Another anesthetist managed anesthesia in the operating theater. An independent research assistant evaluated the nerve block. The study participants and the investigators who performed outcome assessments were blinded to the concentration of the local anesthetic injected and the group assignments during the study period.

### 2.7 Outcome measures

The primary purpose of this study was to determine the effective concentration of ropivacaine through the half-maximal effective concentration (EC50) and the concentration required to achieve the desired effect in 95% of the population (EC95) when combined with varying doses of dexmedetomidine. General patient information was recorded. SBP, DBP, and HR were recorded and analyzed at T0, T1, T2, T3, and T4. The adverse effects and complications after the anesthesia, including hypotension, bradycardia, shivering, pruritus, respiratory depression, nausea, and vomiting, were observed.

### 2.8 Determination of the EC50

We used the Dixon and Massey up-and-down sequential allocation method to determine the EC50 of ropivacaine in each group (19). The first patient in each group received 0.4% ropivacaine for the caudal block (0.75% ropivacaine 10.7 mL + 0.9% normal saline 9.3 mL), along with normal saline or dexmedetomidine administered intranasally. The subsequent concentration of ropivacaine was determined based on the analgesic responses of the previous patients in the same group during the pinprick testing. The patients were asked to report their feelings in response to the pinprick testing of the perineal anal area within 20 min after the caudal block. An effective caudal block—indicated by the absence of pain in response to the pinprick testing of the perineal anal area—meant that the subsequent patient would receive a 0.025% lower dose of ropivacaine. Inversely, an ineffective block, where the patient experienced numbness but still perceived pain in response to the pinprick testing of the perineal anal area, resulted in an increase of 0.025% for the next patient. When a technical failure of the caudal block was identified, this patient was excluded from this study. The next patient received the same concentration as the excluded one. The dosage of EC50 was determined from the midpoints of all independent pairs of patients who were involved in a crossover from “effective” to “ineffective,” and enrolment continued until at least six pairs were obtained.

### 2.9 Sample size calculation

Generally, the up-and-down sequential method stops recruiting patients after six crossovers occur, making it impossible to determine the accurate sample size in advance (20). According to published research (19), the up-and-down allocation method requires 20–40 study participants to estimate the EC50. Based on references (21–23), a sample size of 30 cases per group was selected and considered sufficient to obtain six pairs of reversals in sequence.

### 2.10 Statistical analysis

We conducted a statistical analysis using SPSS version 23 (IBM Corp, Armonk, NY). Demographic data and various intraoperative indicators were collected. Continuous variables with a normal distribution were presented as mean ± standard deviation (SD), while data with an abnormal distribution were presented as median, interquartile range (IQR), and range. Categorical variables were expressed as percentages. The data for the continuous variables with a normal distribution were analyzed using one-way ANOVA, and the least significant difference (LSD) method was applied for multiple comparison tests between the groups. The data for the non-normally distributed variables were compared using Kruskal–Wallis one-way ANOVA. Counts were analyzed using the chi-square (χ^2^) test. The EC50 of ropivacaine in each group was estimated using the Dixon and Massey up-and-down sequential allocation method and probit regression. Bilateral tests were performed for all tests, and a *p* value of <0.05 was considered indicative of significant differences.

## 3 Results

**Figure 3:**
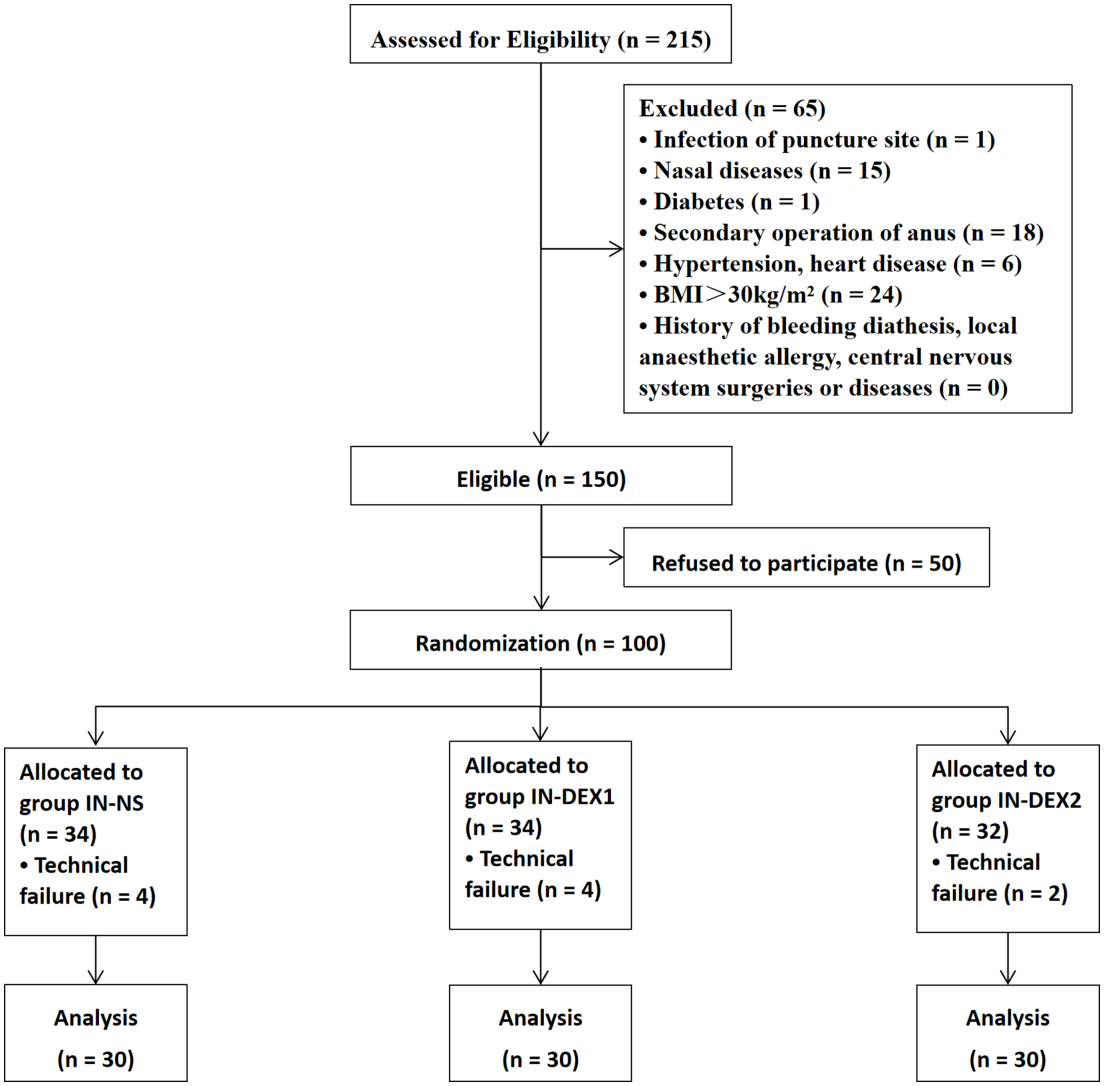
Trial flow chart.

A total of 100 patients were recruited, randomized, and received sacral anesthesia from 1 May 2018 to 6 January 2019. A total of 10 patients were excluded because of technical failures, which included four cases of punctured vessels, four cases of unilateral blocks, and two cases of no anesthetic effect from the caudal block. Ultimately, 90 patients were included in this study and divided into three groups (30 patients in each group) (Figure 3).

### 3.1 Demographic data and clinical characteristics

There were no significant differences in age, sex, height, weight, ASA classification, caudal block operation time, and surgery duration among the different groups (*p* > 0.05) (Table 1).

**Table 1:** Demographic data and operation time.

	Group IN-NS (*n* = 30)	Group IN-DEX1 (*n* = 30)	Group IN-DEX2 (*n* = 30)
Age (years, mean ± SD)	37.1 ± 7.5	38.1 ± 7.1	35.9 ± 8.5
Gender (Male/female)	14/16	12/18	13/17
Weight (kg)	60.5 ± 10.4	58.3 ± 10.8	60.0 ± 11.1
Height (cm)	164.7 ± 7.1	163.9 ± 7.1	164.7 ± 9.1
ASA (grade, I/II)	19/11	19/11	19/11
Operation time of caudal block (min, mean ± SD)	6.3 ± 1.5	6.1 ± 1.4	6.4 ± 1.7
Operation time of surgery (min, mean ± SD)	34.0 ± 10.4	33.7 ± 12.5	38.8 ± 11.6

Data were presented as mean ± SDs or count.

### 3.2 Median effective concentration

**Figure 4:**
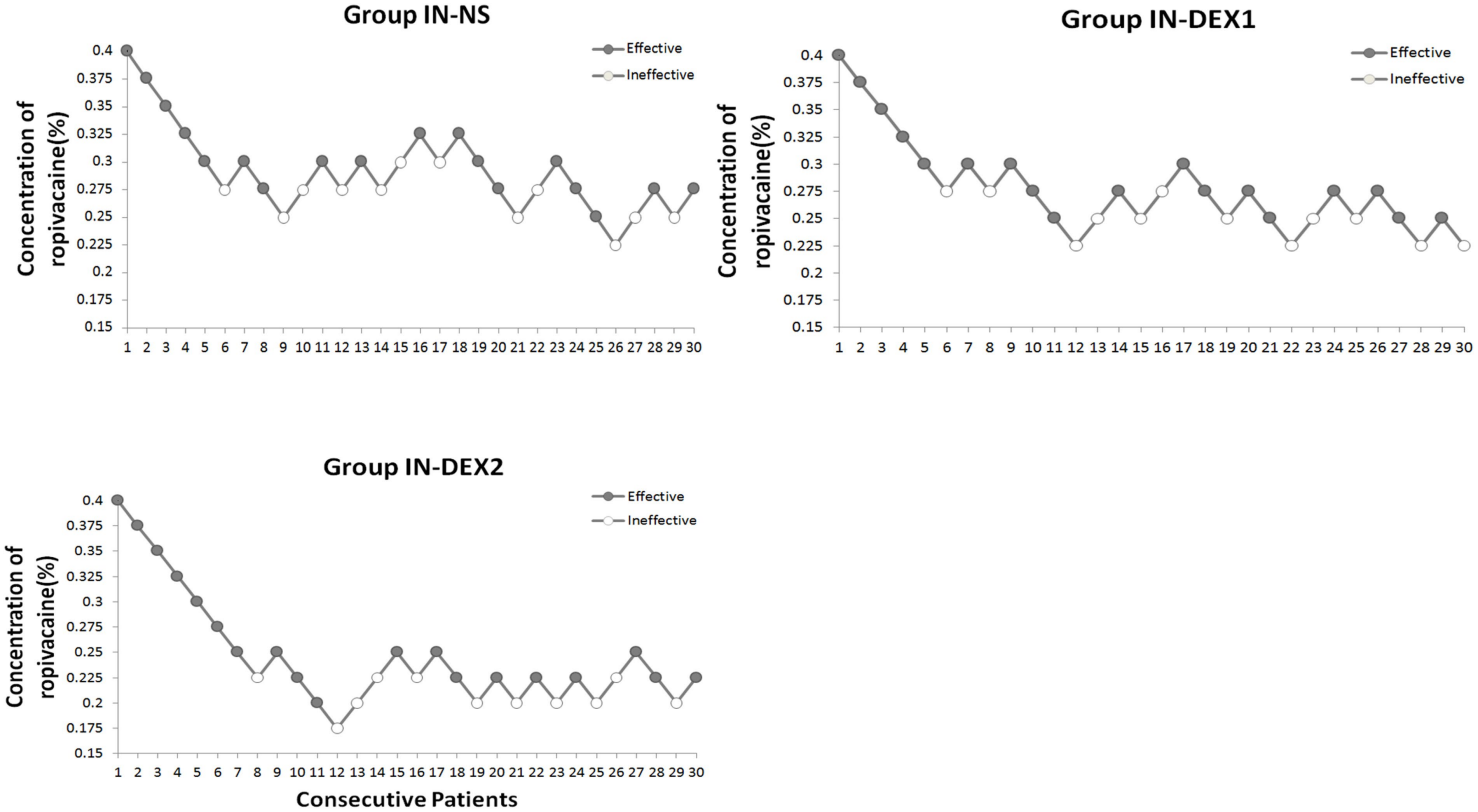
Dose–response concentrations of ropivacaine for caudal block using the up-and-down method in the study groups. The testing interval was 0.025%, “●,” an effective analgesia. “〇,” an ineffective analgesia. Group IN-NS, IN-DEX1, and IN-DEX2 were all the administered intranasally, the sequential concentrations of caudal ropivacaine with intranasal 0.9% sodium chloride solution in group IN-NS, and the sequential concentrations of caudal ropivacaine with 1 μg/kg and 2 μg/kg dexmedetomidine intranasally in group IN-DEX1 and IN-DEX2, respectively.

Figure 4 shows the sequences of the success and failure outcomes using the up-and-down sequential allocation method. The EC50 values of ropivacaine for the caudal block were 0.275% [95% confidence interval (CI), 0.254–0.296%] in group IN-NS, 0.257% (95% CI, 0.238–0.276%) in group IN-DEX1, and 0.216% (95% CI, 0.195–0.236%) in group IN-DEX2. (*p* < 0.05) (Table 2). The EC95 values of ropivacaine were 0.315% (95% CI, 0.295–0.370%) in group IN-NS, 0.297% (95% CI, 0.278–0.351%) in group IN-DEX1, and 0.256% (95% CI, 0.236–0.310%) in group IN-DEX2. Compared to group IN-NS, the EC50 value of ropivacaine in group IN-DEX2 was significantly decreased by 21.4% (*p* = 0.001), while the EC50 value of ropivacaine in group IN-DEX1 did not show a significant decrease (*p* = 0.125).

**Table 2:** Dose–response data of ropivacaine for caudal block in the study groups derived by the Dixon–Massey up-and-down sequential allocation method and probit regression.

	Group IN-NS (*n*)	Group IN-DEX1 (*n*)	Group IN-DEX2 (*n*)	*p* value
Dixon–Massey EC50, %	0.275 (95%CI, 0.254–0.296) (16)	0.257 (95%CI, 0.238–0.276) (18)	0.216^a^^b^ (95%CI, 0.195–0.236) (16)	0.000
Probit regression EC50, %	0.277 (95%CI, 0.263–0.290) (30)	0.259 (95%CI, 0.245–0.273) (30)	0.218^a^^b^ (95%CI, 0.203–0.232) (30)	0.036

Data were EC50 with 95% CI. *p* < 0.05 was considered to indicate significant differences.

a *p < 0.05, compared with the group IN-NS.*

b *p < 0.05, compared with the group IN-DEX1.*

### 3.3 Hemodynamics

There were significant differences in hemodynamic changes between the groups depending on whether dexmedetomidine was used (Table 3). Compared to group IN-NS, SBP and DBP at T3 and T4, as well as HR at T4, were significantly lower in group IN-DEX1 (*p* < 0.05), while SBP, DBP, and HR at T3 and T4 were significantly lower in group IN-DEX2 (*p* < 0.05). Compared to group IN-DEX1, HR at T3 and T4 was significantly lower in group IN-DEX2 (*p* < 0.05).

**Table 3:** Comparisons of blood pressure and heart rate levels among groups.

	Group IN-NS (*n* = 30)	Group IN-DEX1 (*n* = 30)	Group IN-DEX2 (*n* = 30)	*p* value
Systolic blood pressure				
T0	124.3 ± 11.3	122.7 ± 12.7	121.4 ± 9.9	0.606
T1	121.0 ± 8.6	120.0 ± 8.6	118.6 ± 7.3	0.524
T2	117.8 ± 7.7	117.5 ± 7.7	115.4 ± 6.1	0.396
T3	122.2 ± 9.1	114.0 ± 7.4^a^	111.0 ± 6.4^a^	0.000
T4	118.9 ± 7.7	109.7 ± 9.6^a^	106.6 ± 7.4^a^	0.000
Diastolic blood pressure				
T0	78.5 ± 8.6	78.4 ± 7.9	76.9 ± 7.1	0.673
T1	76.3 ± 7.5	75.5 ± 6.3	75.9 ± 6.4	0.884
T2	75.2 ± 5.8	74.3 ± 5.7	73.6 ± 6.4	0.585
T3	77.5 ± 6.9	71.8 ± 6.5^a^	69.2 ± 6.8^a^	0.000
T4	76.0 ± 6.1	67.9 ± 8.0^a^	64.2 ± 6.4^a^	0.000
Heart rate				
T0	77.4 ± 9.7	77.3 ± 7.5	76.8 ± 8.3	0.964
T1	75.4 ± 7.5	75.4 ± 6.1	74.9 ± 7.6	0.955
T2	74.2 ± 7.9	73.3 ± 6.5	70.6 ± 6.8	0.129
T3	74.5 ± 8.8	71.0 ± 7.8	65.1 ± 7.3^a^^b^	0.000
T4	74.1 ± 8.4	68.1 ± 7.6^a^	62.6 ± 7.2^a^^b^	0.000

Data were presented as mean ± SDs. *p* < 0.05 was considered to indicate significant differences.

T0, Baseline; T1, 0 min before drug adminstration; T2, 0 min before caudal block; T3, 5 min after caudal block; and T4, 20 min after caudal block.

a *p < 0.05, compared with the group IN-NS.*

b *p < 0.05, compared with the group IN-DEX1.*

### 3.4 Side effects

There were several cases of hypotension and bradycardia in both groups IN-DEX1 and IN-DEX2; however, we did not find any significant differences among the three groups. In addition, there were no statistical differences in the incidence of other adverse effects between the groups, either (Table 4).

**Table 4:** Adverse effects among groups.

	Group IN-NS (*n* = 30)	Group IN-DEX1 (*n* = 30)	Group IN-DEX2 (*n* = 30)	*p* value
1. Nausea and vomiting (*n*)	2	3	4	0.157
2. Shivering (*n*)	0	0	0	1.000
3. Pruritus (*n*)	0	0	0	1.000
4. Hypotension with intervention (*n*)	0	0	1	0.221
5. Bradycardia with intervention (*n*)	0	1	2	0.157
6. Hypertension with intervention (*n*)	0	0	0	1.000
7. Respiratory depression (*n*)	0	0	0	1.000

Data were presented as number.

## 4 Discussion

The effect of a caudal block is influenced by the volume and concentration of local anesthetics; however, high concentrations of local anesthetics often lead to many adverse consequences. Therefore, it is important to reduce the concentration of local anesthetics while ensuring the effect. In this study, the intranasal route of dexmedetomidine reduced the EC50 of ropivacaine used in a caudal block and demonstrated a dose-dependent effect. Given that nasal administration is simple and well-accepted by patients, IN-DEX is worthy of clinical application.

Ropivacaine is a commonly used analgesic for caudal blocks due to its fast onset and long-acting properties. Some studies have indicated that the concentration of ropivacaine for a caudal epidural block ranges from 0.2 to 0.5% (24). In a double-blind prospective study on the concentration of ropivacaine administered for ultrasound-guided caudal epidural blocks, the minimum effective concentration (MEC95) was found to be 0.362% (95% CI, 0.322%–0.612%) for 20 mL of ropivacaine (25). In this study, we chose 0.4% ropivacaine as the initial concentration to prevent inadequate anesthesia and ensure a reliable concentration. We found that the EC50 of ropivacaine for the caudal block was 0.275% (95% CI, 0.254%–0.296%) in group IN-NS. This finding is consistent with a previous study by Ma et al., in which the authors found that the MEC50 for a caudal epidural block of ropivacaine at 20 mL was 0.276% (95% CI, 0.236%–0.308%) (25).

Numerous studies have confirmed that combining adjuvants with local anesthetics can prolong the duration of action and reduce the dosage of anesthetics (26). Our research also demonstrated that the EC50 was reduced in the IN-DEX group. Dexmedetomidine used as an adjuvant to local anesthesia can synergize with local anesthetics, thereby shortening the onset time of anesthesia, prolonging analgesia, and reducing the dosage of anesthetics (27–29). The mechanism of dexmedetomidine as an adjuvant for analgesia includes both central and peripheral analgesic effects. The mechanism of peripheral analgesia primarily involves the activation of α2 adrenoceptors in peripheral blood vessels, leading to peripheral vascular contraction, which delays the absorption of local anesthetics and extends the action time (30). In addition, the central mechanism primarily involves the action on brainstem α2 adrenergic receptors, which inhibit the medullary noradrenergic pathway and terminate the propagation of pain signals, leading to analgesia (31).

Compared to the intravenous route, the intranasal route is more accessible because of its non-invasive systemic administration (18, 32). Dexmedetomidine has a molecular weight of 236.7 Dalton and is easily absorbed through the nasal mucosa. After intranasal absorption, dexmedetomidine enters the central nervous system through the blood–brain barrier and exerts pharmacological effects similar to intravenous administration (33, 34). The difference lies in the fact that the onset time of IN-DEX is slower than that of IV-DEX; this more gradual onset helps avoid the α_1_-adrenoreceptor agonist effects associated with rapid intravenous access (hypertension and bradycardia) (33, 34). Many studies have shown that IN-DEX is safer, more effective, and more comfortable and convenient than IV-DEX. A systematic review by Poonai et al. reported that the onset times of intranasal dexmedetomidine were inconsistent and ranged from 7 to 31 min (35). Therefore, we designated the pretreatment time for intranasal dexmedetomidine as 15 min before the caudal block, in addition to the time of the caudal block procedure, which required 15–20 min. Previous findings suggested that patients receiving IN-DEX experienced better postoperative pain relief (36) and reduced use of analgesics (37). However, there are few studies on the effects of IN-DEX on regional anesthesia. A prospective, randomized, double-blind trial on nulliparous patients found that pretreatment with IN-DEX before epidural labor analgesia provides a quicker onset of analgesia and decreases epidural puncture pain without increasing adverse effects (38). However, no data on the efficacy and safety of IN-DEX as an adjuvant for caudal blocks are available. A pilot study was conducted with two doses (1.0 and 2.0 μg/kg), in which 2.0 μg/kg was found to be the optimal dose based on a previous study of IN-DEX for perioperative sedation and analgesic treatment in adults (39). Our study also confirmed that IN-DEX decreased the EC50 of ropivacaine for the caudal block, showing that 2 μg/kg of IN-DEX reduced the EC50 of ropivacaine by 21.4%. While 1 μg/kg of IN-DEX also reduced the EC50 of ropivacaine, there was no statistically significant difference between group 1 μg/kg IN-DEX and group IN-NS, which might have been a result of bioavailability. In addition, this study demonstrated a specific dose-dependent correlation between IN-DEX and the EC50 of ropivacaine for the caudal block, which was similar to previous studies (40).

The caudal block using the blind puncture technique based on anatomical localization markers has a specific failure rate due to anatomical variations, especially the sacral hiatus and sacral cornual variations. This traditional blind puncture technique can be challenging for adult patients; only a 75% success rate has been reported (41). Ultrasonography is useful for visualizing the sacral hiatus and sacrococcygeal ligament, which helps improve the success rate of the puncture. Our success rate for the caudal block was 90.0%, which was lower than the very high success rates (96.9%–100%) reported in various studies for ultrasound-guided caudal epidural blocks (42, 43). The main reason for this is that we adopted pre-puncture ultrasonic positioning instead of real-time ultrasound-guided puncture. Although pre-puncture positioning can effectively identify the puncture point and needle insertion depth, helping the operator find the best puncture path, it is difficult to completely follow the “perfect” needle insertion path during the subsequent blind puncture process. As aseptic technique is required for real-time ultrasound guidance, it takes more time for the aseptic preparation of the probe. Given the high volume of anorectal procedures performed daily at our hospital, which necessitates quick turnover, we opted for ultrasonic positioning.

Decreased blood pressure and heart rate are common side effects of dexmedetomidine. Compared to the IN-NS group, both blood pressure and heart rate decreased significantly at T3 and T4 in the IN-DEX group. However, there was no statistical difference between the hypotension and bradycardia groups requiring medical intervention. At the same time, we found that compared to our previous study, the decrease caused by intravenous dexmedetomidine in blood pressure and heart rate was lower. This finding is consistent with the study by SINGLA et al., who compared hemodynamic responses between intravenous and nebulized dexmedetomidine (1 μg/kg) and found that blood pressure was significantly lower in group IV as compared to group IN (44). This may be due to the slower drug absorption after intranasal dexmedetomidine, which causes the peak concentration to be significantly lower than that of its intravenous counterpart (45). In our study, there were several cases of hypotension and bradycardia that required drug treatment. However, there was no statistically significant difference between the groups, which might have been influenced by the limited sample size of this study.

## 5 Limitations of this study

In this study, we observed and compared the effects of IN-DEX on the EC50 of ropivacaine for caudal blocks. The participants were screened strictly according to the inclusion criteria. However, the study still has some limitations. Firstly, gender was not considered a confounding factor in this study, which may be a limitation. It has been reported that there are gender differences in the pharmacokinetics and pharmacodynamics of some anesthetics (46). Moreover, Asghar et al. reported that men have higher volumes of the sacral canal and caudal space compared to women (47). However, Pei et al. (48) found that gender did not affect the EC50 of ropivacaine in nerve blocks. Therefore, further studies are needed to determine whether gender could influence the synergistic effect of dexmedetomidine on ropivacaine for caudal blocks. In addition, based on our previous observations at our hospital, the largest population of patients with hemorrhoids among adults over 18 years old was in the age group of 20–50. To further reduce the impact of age on the study and better evaluate the role of dexmedetomidine, we selected this specific group. Consequently, it remains unclear whether the study results are applicable to the elderly and children. Secondly, for the judgment of pain, this study used the absence of pain in response to the pinprick testing of the perineal anal area as the criterion for determining a successful block. It is different from the study by Li et al. (24), which used the disappearance of perineal skin pain and the relaxation of the anal sphincter as the judgment criteria. Anal sphincter relaxation often indicates an optimal caudal block, but it relies on the surgeon’s judgment as the relaxation of the perianal muscles may differ depending on the opinion of the surgeon. We also found that some patients experienced a complete analgesic effect with poor sphincter relaxation after a caudal block. In clinical practice, such patients generally do not require additional treatment and can successfully complete the operation. Thirdly, dexmedetomidine delivered as nasal drops rather than nasal sprays might have influenced this study. There are some controversies regarding the effect of dexmedetomidine delivered as nasal drops versus nasal sprays on patients. A study found that the sedative effect of dexmedetomidine nasal drops was better than that of dexmedetomidine nasal sprays (49). Conversely, another study did not show a significant difference in bioavailability between nasal drops and nasal sprays, and the sedative effects were similar (34). Therefore, further research is needed to determine whether dexmedetomidine nasal drops and nasal sprays differ in their effects on decreasing the EC50 of ropivacaine for a caudal block. Fourthly, to compare the effects of dexmedetomidine on BP and HR among the groups, this study only selected a period before the surgery for observation to eliminate the influence of various factors as much as possible. However, the changing concentrations of ropivacaine because of the up-and-down sequential allocation method may be a confounding factor. Fifthly, according to relevant studies (12), the common dose of dexmedetomidine as an adjuvant is 0.5–2 μg/kg. Therefore, this study selected doses of 1 μg/kg and 2 μg/kg for research and presented a specific dose-dependent correlation between IN-DEX and the EC50 of ropivacaine for the caudal block. However, we did not investigate larger doses, so the optimal dose needs to be further studied. Finally, the cases of technical failures in the caudal block might have impacted the subsequent process and randomization and might have influenced the research results.

## 6 Conclusion

IN-DEX decreased the EC50 of ropivacaine for the caudal block, and there was a specific dose-dependent effect for IN-DEX. The main side effects of dexmedetomidine included decreased blood pressure and heart rate. However, there were no significant differences in hypotension and bradycardia between the different groups regarding treatment.

## Data Availability

The raw data supporting the conclusions of this article will be made available by the authors, without undue reservation.
